# Motivating landowners to recruit neighbors for private land conservation

**DOI:** 10.1111/cobi.13294

**Published:** 2019-02-20

**Authors:** R. M. Niemiec, R. Willer, N. M. Ardoin, F. K. Brewer

**Affiliations:** ^1^ Emmett Interdisciplinary Program in Environment and Resources Stanford University 473 Via Ortega Way, Suite 226 Stanford CA 94305 U.S.A.; ^2^ Department of Sociology Stanford University Stanford CA 94305 U.S.A.; ^3^ Graduate School of Education & Woods Institute for the Environment Stanford University 485 Lausen Mall Stanford CA 94305 U.S.A.; ^4^ Big Island Invasive Species Committee, Pacific Cooperative Studies Unit University of Hawaii–Manoa 23 E Kawili Street Hilo HI 96720 U.S.A.

**Keywords:** collective action, conservation behavior, Hawaii, invasive, norms, psychology, acción colectiva, comportamiento de conservación, Hawái, normas, psicología, 保护行为, 生物入侵, 集体行动, 夏威夷, 心理学, 规范

## Abstract

Encouraging motivated landowners to not only engage in conservation action on their own property but also to recruit others may enhance effectiveness of conservation on private lands. Landowners may only engage in such recruitment if they believe their neighbors care about the conservation issue, will positively respond to their conservation efforts, and are likely to take action for the conservation cause. We designed a series of microinterventions that can be added to community meetings to change these beliefs to encourage landowner engagement in recruitment of others. The microinterventions included neighbor discussion, public commitment making, collective goal setting, and increased observability of contributions to the conservation cause. In a field experiment, we tested whether adding microinterventions to traditional knowledge‐transfer outreach meetings changed those beliefs so as to encourage landowners in Hawaii to recruit their neighbors for private lands conservation. We delivered a traditional outreach meeting about managing the invasive little fire ant (Wasmannia auropunctata) to 5 communities and a traditional outreach approach with added microinterventions to 5 other communities. Analysis of pre‐ and post‐surveys of residents showed that compared with the traditional conservation outreach approach, the microinterventions altered a subset of beliefs that landowners had about others. These microinterventions motivated reputationally minded landowners to recruit and coordinate with other residents to control the invasive fire ant across property boundaries. Our results suggest integration of these microinterventions into existing outreach approaches will encourage some landowners to facilitate collective conservation action across property boundaries.

## Abstract 2

*Article impact statement*: Social psychology theory can inform outreach that encourages landowners to influence neighbors to participate in conservation.

## Abstract (es)

Motivaciones para que los Terratenientes Recluten a sus Vecinos para la Conservación Privada de Tierras

**Resumen**

Si se alienta a los propietarios motivados a no sólo participar con acciones de conservación en sus propiedades sino también a reclutar a otros, se podría mejorar la efectividad de la conservación en las propiedades privadas. Puede que los propietarios sólo se comprometan con el reclutamiento si consideran que a sus vecinos les importan los temas de conservación, si responderán positivamente a sus esfuerzos de conservación, y si tienen probabilidad de tomar acción por la causa de conservación. Diseñamos una serie de microintervenciones que pueden añadirse a las juntas comunitarias para cambiar estas creencias y así promover la participación de los propietarios en el reclutamiento de otros propietarios. Las microintervenciones incluyeron discusiones entre vecinos, firmas públicas de compromisos, el establecimiento de objetivos colectivos, y una observación incrementada de las contribuciones a la causa de la conservación. En un experimento de campo probamos si la suma de estas microintervenciones a las tradicionales juntas de participación con transferencia de conocimiento cambó dichas creencias de tal manera que alentara a los terratenientes en Hawái a reclutar a sus vecinos para la conservación de terrenos privados. Realizamos una junta tradicional de participación sobre el manejo de la hormiga de fuego (Wasmannia auropunctata), una especie invasora, para cinco comunidades y una estrategia tradicional de participación con la suma de microintervenciones para otras cinco comunidades. El análisis previo y posterior a las encuestas realizadas a los residentes mostró que, si se comparan con la estrategia tradicional de participación, las microintervenciones alteraron a un subconjunto de creencias que los propietarios tenían sobre los demás propietarios. Estas microintervenciones motivaron a los propietarios con una reputación de estar dispuestos a conservar a reclutar y coordinarse con otros residentes para controlar a la hormiga invasora atravesando los límites de las propiedades. Nuestros resultados sugieren que la integración de estas microintervenciones dentro de las estrategias existentes de participación alentará a algunos propietarios a facilitar las acciones de conservación colectiva a través de los límites de las propiedades.

## 【摘要】

鼓励积极的土地所有者不仅在自己的土地上开展保护行动, 还去动员其他人参与保护, 可以提高私有土地的保护成效。然而, 土地所有者只有在相信他们的邻居也关心保护问题、会积极地响应保护工作, 且有可能采取保护行动的前提下, 才会对他们发起动员。我们设计了一系列可以加入到社区会议中的微干预措施, 来改变土地所有者的信念, 鼓励他们参与动员其他人。这些微干预措施包括邻居间的讨论、制定公共承诺、设立集体目标, 提高了对保护贡献的可观察性。在一项实地研究中, 我们测试了在传统的知识传递推广会议中加入这些微干预措施是否能改变这些信念, 从而鼓励夏威夷的土地所有者去动员他们的邻居进行私有土地保护。我们在五个社区开展了关于入侵小火蚁 (Wasmannia auropunctata) 治理的传统推广会议, 而在另外五个社区, 还在传统推广会议中加入了微干预措施的内容。对会议前后的居民调查显示, 与传统的保护推广方法相比, 微干预改变了土地所有者对他人的部分观念。这些微干预措施激励了有声誉意识的土地所有者去动员和协调其他居民, 一起跨越私有土地边界控制入侵火蚁。我们的结果表明, 将这些微干预纳入现有的推广方法中, 可以鼓励土地所有者去推动跨私有土地边界的集体保护行动。**【翻译: 胡怡思; 审校: 聂永刚】**

## Introduction

Achieving conservation objectives across private lands requires that residents engage in conservation actions (Moon & Cocklin 2011). Programs to motivate such actions provide landowners with education, technical assistance, management supplies, financial incentives, and certifications (Moon & Cocklin 2011; Ma et al. 2012). To recruit landowners for these programs, organizations typically teach landowners about the economic and ecological benefits of the program and how to participate (knowledge‐transfer approach) (Ma et al. 2012). Such approaches often do not motivate widespread engagement in conservation activities on private land (Ma et al. 2012). Despite decades of outreach, only 2% of U.S. family–forest owners have an easement on their land, 6% participate in cost‐share programs, and 1% participate in forest certification programs (Butler 2008). Outreach focused on invasive species management on private lands has not motivated widespread landowner action in Australia (Graham 2013) or the United States (Ma et al. 2018). Such efforts often motivate only private landowners who are already generally committed to active management, have participated in extension events, and have adopted conservation practices (Langer 2008; Ma et al. 2012). These motivated individuals are referred to as “model landowners” (Langer 2008).

The body of research on how to expand private lands conservation (Snyder & Broderick 1992; Ma et al. 2012) suggests peer‐learning approaches are an effective strategy; model landowners teach other community members about the conservation program and encourage them to participate (Snyder & Broderick 1992; Kueper et al. 2013). Motivating model landowners to communicate with others in their community may increase the reach of conservation programs. Peer interactions may create community social norms around a conservation behavior (McKiernan 2017), which could facilitate conservation action over time (Cialdini et al. 2003; Niemiec et al. 2016). They may enhance landowner feelings of efficacy and reduce concerns about the program, encouraging widespread participation (Snyder & Broderick 1992; Bandura 1998). Model landowners may be more successful than program staff at convincing others to participate in a conservation program because they may be more trustworthy and credible due to shared experiences and values (Rogers 2003; Gootee et al. 2010). Additionally, achieving conservation objectives often requires landowners to coordinate across property boundaries (Graham & Rogers 2017; Ma et al. 2018). Reducing the spread of invasive species, for example, may require landowners to simultaneously apply control tactics across properties (Graham & Rogers 2017). Enhancing habitat connectivity may require that landowners work together to ensure a sufficient corridor of habitat across multiple properties.

Together, this literature suggests that to enhance the reach and effectiveness of private lands conservation, outreach programs should encourage model landowners to share information, recruit, and facilitate coordinated efforts with their neighbors. However, researchers know little about how to motivate model landowner engagement in such collective practices. Few model landowners teach, recruit, or coordinate with their neighbors for conservation purposes (Niemiec et al. 2016; Ma et al. 2018; Niemiec et al. 2018), and people appear unwilling to discuss environmental issues, even with close friends or family (Geiger & Swim 2016). It remains unclear how to encourage model landowners to move beyond focusing on their own property to reaching out to their neighbors to further conservation causes.

The behavioral‐science literature suggests that when sharing information, recruiting, or coordinating with others for collective action, people may be influenced by the perceived actions and beliefs of relevant others (Simpson & Willer 2015). Individuals appear more willing to recruit and work with others to achieve a collective goal when social norms support the behavior (Niemiec et al. 2016), when individuals believe others will also take action (Simon et al. 1998; Niemiec et al. 2016), and when individuals believe they can receive social or reputational rewards by engaging in collective action (Simon et al. 1998). Changing perceptions of others’ beliefs can encourage discussion of environmental issues with others (Geiger & Swim 2016). Supplementing current outreach approaches with interventions that alter such beliefs about others may motivate model landowners to communicate with their neighbors.

From the literature, we identified microinterventions focused on altering beliefs about others to enhance model landowner engagement in collective action. We call them microinterventions because they are simple, low‐cost additions to existing outreach and educational programs (in this case community meetings). The first hypothesis we tested is that adding social‐psychologically based microinterventions to traditional outreach approaches (i.e., knowledge transfer) more effectively brings about positive change in model landowners’ beliefs about others’ attitudes and actions toward the conservation behavior. The second hypothesis we tested is that adding microinterventions to traditional outreach approaches increases model landowners’ engagement in peer sharing, recruitment, and coordination with their neighbors (hereafter, recruitment and coordination behavior) for private lands conservation.

We also examined how the microinterventions might influence within‐property conservation behaviors. Studies show mixed results on whether perceptions of others may influence personal conservation behaviors (e.g., Cialdini et al. 2003; Niemiec et al. 2018). Further, a trade‐off may exist between encouraging recruitment and coordination and encouraging property‐level contributions if landowners have limited time and resources. We therefore also examined whether our microinterventions may influence property‐level conservation action.

## Methods

### Microinterventions and Targeted Beliefs

The microinterventions focused on changing perceived descriptive and injunctive norms, reputational incentives, expected reciprocity, and collective efficacy (Table 1) by facilitating increased communication among model landowners attending an outreach meeting about their experiences with the conservation challenge (Lewin 1947); encouraging model landowners attending an outreach meeting to publicly commit to recruit and coordinate with others (Lewin 1947; Lokhorst et al. 2013); facilitating model landowner development of community‐specific collective goals related to conservation (Bandura & Schunk 1981; Mitkidis et al. 2013); and increasing visibility of model landowners’ contributions to conservation after the outreach meetings (Simpson & Willer 2008; Yoeli et al. 2013). We chose these microinterventions because of their demonstrated impact on beliefs about others and the ease with which they can be integrated into an outreach meeting structure.

**Table 1:** How microinterventions may influence model landowners’ beliefs about others. Microinterventions

	Enhanced communication among neighbors at community meeting about conservation problem	Collective goal setting at meeting	Public commitment making at community meeting	Enhanced visibility of contributions after meeting through yard signs and postcards
Social Perception Expected reciprocity and collective efficacy	By learning that others care about the conservation problem through discussion, model landowners may come to believe that others will respond positively to their recruitment efforts and that enough others will contribute to achieve a collective goal.	Ascribing and then achieving proximal goals can enhance efficacy beliefs; developing, visualizing, and achieving specific proximal collective goals related to the conservation problem may enhance model landowners’ collective efficacy beliefs related to the conservation cause (Bandura & Schunk 1981).	Viewing others’ public commitments to the conservation cause may convince model landowners others will take action to fulfill their commitments. They may start to believe enough others can be inspired to contribute to achieve a collective conservation goal.	Seeing others start to contribute to the conservation problem may help convince model landowners they are not alone in their actions; rather, model landowners may start to believe that enough others can be inspired to contribute to achieve a collective goal.
Descriptive and injunctive norms	Neighborhood discussion may enhance belief that other community members care about the conservation cause and are engaging in collective conservation efforts; this enhanced descriptive and injunctive norm may make model landowners more comfortable approaching others about the issue (Geiger & Swim 2016).	Collective goal setting may help develop shared understandings that engaging in recruitment and coordination behavior is important and valued to achieving an agreed‐upon collective goal; these shared understandings (i.e., injunctive norms) may make model landowners more comfortable approaching others (Geiger & Swim 2016).	Seeing others’ public commitments may enhance belief that others in the community care about the conservation cause and are engaging in collective conservation efforts; this enhanced descriptive and injunctive norm may make model landowners more comfortable approaching others about the issue (Geiger & Swim 2016).	Viewing others’ positive efforts can lead to a new community norm that people are engaging in and care about the desired conservation behavior, which may make model landowners more comfortable approaching others (Hopper & Nielsen 1991).
Reputational rewards or sanctions	Neighborhood discussion can highlight how much other landowners care about whether one engages in recruitment and coordination for the conservation cause; it can lead to the perception that model landowners are likely to get rewards (or sanctions) from others for engaging (or not) in recruitment and coordination behavior.	Collective goal setting can highlight how much other landowners care about whether one engages in recruitment and coordination for the conservation cause; it can lead to the perception that model landowners are likely to get rewards (or sanctions) from others for engaging (or not) in recruitment and coordination behavior.	Public commitments motivate behavior in part by creating social pressure to uphold one's public image (Lokhorst et al. 2013). Model landowners who publicly commit to recruitment and coordination may feel pressure to engage in such behavior to uphold their reputation.	Enhancing the visibility of people's potential actions can effectively invoke the power of reputational incentives (Simpson & Willer 2008; Yoeli et al. 2013). Making model landowner's contributions to the collective conservation problem visible allows for model landowners to potentially obtain reputational rewards for contributing to the conservation cause.

We based the microinterventions on research on injunctive and descriptive social norms. Injunctive social norms refer to perceived social approval or disapproval associated with a behavior, whereas descriptive social norms refer to perceptions of how commonly others practice a behavior (Cialdini et al. 2003). Such normative beliefs may influence recruitment and coordination behavior. Individuals appear more willing to reach out to others when they believe others are also concerned about the issue (i.e., there are strong “injunctive norms” around the issue) (Geiger & Swim 2016; Niemiec et al. 2016). Interventions demonstrating that others are also concerned increase people's willingness to approach others about climate change (Geiger & Swim 2016).

We also drew on research suggesting that individuals with high levels of efficacy are more likely to engage and persist in undertaking collective action (Bandura 1998; Geiger et al. 2017). Two types of efficacy are particularly relevant for recruitment and coordination. Expected reciprocity suggests individuals are more likely to approach others when they believe their actions will inspire others to contribute (Lubell et al. 2007; Niemiec et al. 2016; Geiger et al. 2017). Collective efficacy suggests people are more likely to work with others when they perceive a group has the capacity to work together to achieve a collective goal (Bandura 1998).

The microinterventions were also drawn from the role of reputational incentives in influencing collective action (Simpson & Willer 2008). The desire to be viewed positively by others and avoid others’ negative perceptions is strongly motivating. In situations where others can notice an individual's collective contributions, those individuals may be more likely to contribute (Yoeli et al. 2013; Simpson & Willer 2015).

Building on Lewin (1947), we incorporated increased communication and public commitment making as microinterventions to change normative perceptions about which others care about and are engaging in conservation action. By learning that others care about the conservation cause through these strategies, community members may come to believe that others will respond positively to their recruitment efforts (i.e., have greater expected reciprocity), that the group might achieve a collective goal (i.e., have greater collective efficacy), and that others would react with reputational rewards (or sanctions) if residents did (or did not) engage in recruitment and coordination behavior.

We incorporated community‐specific collective goals at outreach meetings to increase perceived efficacy, building on social psychology research demonstrating the role of goal setting in enhancing efficacy and cooperation (Bandura & Schunk 1981; Mitkidis et al. 2013). We included increased visibility of contributions through yard signs and postcards to create the possibility for reputational rewards by making visible residents’ collective contributions after meetings. The yard signs were also meant to develop a descriptive norm signaling that others are engaging in the conservation cause, while the enhanced neighbor‐to‐neighbor communication as a result of the yard signs and postcards was meant to enhance injunctive norms, suggesting that others care about the conservation cause (Hopper & Nielsen 1991).

### Study Context

We tested our microinterventions where a growing conservation threat required widespread landowner action and coordination, a conservation organization wanted to expand outreach beyond model landowners to facilitate widespread engagement, and model landowner engagement in recruitment and coordination with neighbors for conservation was uncommon. We focused on the management of the invasive little fire ant (LFA) (*Wasmannia auropunctata*) across private lands on Hawaii. This species has also invaded Melanesia, Galapagos Islands, Florida, and possibly California (U.S.A.), Bahamas, Bermuda, and parts of West Africa (Wetterer & Porter 2003). In Hawaii, residents and entomologists first detected LFA in 1999. It is now widespread along the island's eastern coast. Little fire ant can displace native ant populations, interfere with bird nesting, cause blindness in animals, and facilitate growth of agricultural‐pest populations (Wetterer & Porter 2003).

Widespread, coordinated resident actions are essential to limit further spread and reduce current levels of LFA in Hawaii (Lee et al. 2015). To check their property for infestation (Vanderwoude et al. 2010), residents use a peanut butter bait. If LFA is present, residents repeat baiting‐and‐barrier treatments every 4–6 weeks for up to 1 year. The LFA may move among properties, so coordinating with neighbors to apply the same treatments over multiple properties can enhance treatment effectiveness.

Because of the growing threat of LFA, in 2016, prior to our experiment, the Big Island Invasive Species Committee (BIISC) planned an outreach campaign to motivate residents to manage LFA. The BIISC is a project of the University of Hawaii Pacific Cooperative Studies Unit and represents a voluntary partnership among government, private, and nonprofit organizations. For several years prior to our experiment, BIISC conducted community outreach meetings to encourage private landowners to manage invasive species on their properties. The outreach efforts of BIISC focused on providing technical support, subsidized control supplies, and information on the conservation problem and targeted conservation actions. Residents attending BIISC's meetings usually were already concerned about the problem and engaging in conservation action on their property (Niemiec et al. 2018). The BIISC found it difficult to reach uninvolved community members, and model landowners rarely recruited and coordinated with their neighbors, despite the need for widespread landowner participation (Niemiec et al. 2016; Niemiec et al. 2018). We tested whether microinterventions could motivate model landowners to engage in more recruitment and coordination behaviors, thereby enhancing their conservation achievements across private lands.

### Experimental Design

We used a cluster‐randomized control trial to compare effectiveness of a community workshop with added microinterventions (delivered in 5 intervention communities) to a traditional workshop focused on knowledge transfer (delivered in 5 control communities). In collaboration with BIISC in 2016, we advertised an outreach program to LFA‐infested communities via emails to community leaders, an article in the local newspaper, and BIISC's website. For program enrollment, a self‐designated community leader worked with BIISC to organize a location and date for a community meeting. By the specified deadline, 12 communities had signed up for the program. In 2 communities, we tested and refined various microinterventions. The experiment (including intervention and control groups) focused on individuals in the remaining 10 communities.

To ensure covariate balance between control and intervention communities, we divided the 10 communities into matched pairs based on key characteristics (e.g., median income, percentage of residents over the age of 65, presence or absence of a community association, and level of urbanity) (matching variables described in Supporting Information). Within each matched pair, we randomly assigned 1 community to receive the intervention and 1 to receive the control treatment. The matching and randomization led to overall balance in key covariates between control and intervention groups (Supporting Information).

The BIISC delivered a primarily informational lecture about LFA, including management tactics, at a meeting in all 10 communities. In the 5 intervention communities, we added activities related to the 4 microinterventions (Table 1 & Supporting Information). In total, 211 individuals attended the 10 meetings (range 11–37/meeting).

We conducted 3 surveys to assess impacts. At the meeting, before sharing content, we administered a preprogram survey; 2 and 7 months after the meeting, we administered follow‐up surveys. In the preprogram and 2‐month follow‐up surveys, we included 7 items to assess participants’ perceived social norms, reputational rewards or sanctions, expected reciprocity, and collective efficacy (Supporting Information). In the preprogram and both follow‐up surveys, we included items related to model landowners’ self‐reported engagement in recruitment and coordination and their personal, on‐property conservation efforts. We measured recruitment and coordination behaviors as the sum of the number of times individuals taught others how to control LFA, tried to convince others to control LFA, or organized efforts with neighbors to control LFA. We measured property‐level conservation efforts as the sum of the number of times individuals surveyed their property for LFA, applied commercial or noncommercial products to control LFA on their property, or hired someone to control LFA on their property (Supporting Information).

Surveys also included items about demographics and additional perceptions that could influence contributions to LFA management (e.g., knowledge and risk perceptions) (Supporting Information). To determine whether the microinterventions influenced some residents more than others, we measured self‐reported value orientations in the presurvey (Supporting Information). We measured participant's egoistic, altruistic, and biospheric values and their desired respect from others. Beliefs about others may have a greater impact on more egoistic individuals (i.e., those who lack the strong intrinsic motivation of altruists to contribute to the collective and define their reputation by or in relation to others) (Simpson & Willer 2008). We therefore hypothesized that individuals with high reputational concern and more egoistic values would be more heavily influenced by the microinterventions (Supporting Information).

We used ordinal logistic regression to model the intervention effects on beliefs about others because we measured those beliefs as ordinal Likert‐type survey items; thus, they were not continuous variables. We examined results of linear regression models to assess the sensitivity of results to use of qualitatively different models (Supporting Information). We used a Poisson regression to model the intervention effects on behavioral outcomes because it had the lowest cross‐validation mean squared error of all possible models for count data. We determined the results for negative binomial models to assess the sensitivity of the study results to the use of qualitatively different models (Supporting Information). We conducted a moderation analysis to examine whether the intervention effects differed based on respondents’ value orientations. In all regression analyses, we controlled for preprogram perceptions or behaviors, which varied slightly between intervention and control groups (Table 2), and used robust SEs clustered at the community level (Supporting Information).

**Table 2:** Descriptive statistics from the intervention and control communities before the program survey (*n* = 162^a^)

Variable	Metric	Control mean (n = 79)	Control SD	Intervention mean (n = 83)	Intervention SD
Preprogram behaviors^b^					
recruitment and coordination behavior	sum of 3 behaviors, with maximum of 18 possible	1.84	3.44	1.59	3.06
property‐level behavior	sum of 3 behaviors, with maximum of 18 possible	6.77	5.12	7.65	6.08
Preprogram beliefs about others					
expected reciprocity	7‐point Likert	5.00	1.24	5.36	1.16
collective efficacy 1. sufficient numbers of residents can be mobilized	7‐point Likert	4.90	1.31	5.03	1.26
collective efficacy 2. together, residents can achieve a collective goal	7‐point Likert	5.28	1.34	5.20	1.35
injunctive norms prevalence of LFA concern	7‐point Likert	5.09	1.33	5.28	1.35
descriptive norms prevalence of LFA control behavior	6‐point scale, ranging from 0–100%	2.91	1.14	2.71	1.35
potential for reputational rewards	7‐point scale ranging from –3 (negative reaction) to 3 (positive reaction)	5.49	1.29	5.62	1.22
potential for reputational sanctions	7‐point scale ranging from –3 (negative reaction) to 3 (positive reaction)	3.39	1.05	3.48	1.10
Demographics					
property ownership	1 = yes, 2 = no	1.14	0.344	1.15	0.356
income	5‐point interval scale	2.93	1.43	2.98	1.38
property size (ac)^c^		3.05	6.39	2.44	4.05
education	5‐point interval scale	3.42	1.24	3.49	1.26
Age		57.02	14.82	62.08	13.30
Preprogram perceptual covariates					
threat perceptions	5‐point scale	4.06	0.724	4.07	0.870
knowledge of control tactics	7‐point Likert	3.59	1.98	3.59	2.05

a Mean number of people attending outreach meetings in the control communities was 22, and mean number of people attending outreach meetings in the intervention communities was 20.

b Survey gave residents the option of indicating they had engaged in the behavior 0–6 times; numbers greater than 6 were counted as 6 in the analyses.

c Abbreviation: ac, acres.

## Results

Of the 211 community members who attended the 10 experimental community meetings, we received the following number of completed surveys: 162 preprogram, 89 2‐month follow‐up, and 86 7‐month follow‐up. Preprogram behaviors, beliefs about others, and demographics were similar among intervention and control groups, suggesting randomization achieved relative balance across groups (Table 2). Attrition rates were high, especially between the preprogram and 2‐month follow‐up surveys (Supporting Information). However, on examination of mean baseline characteristics between intervention and control groups (Supporting Information), attrition did not appear to cause differential loss to follow‐up. There were no significant differences in preprogram recruitment and coordination between those who dropped out after the presurvey and those who remained in the study to complete the 2‐ and 7‐month follow‐ups in the intervention and control groups (Supporting Information). We therefore used complete cases for our analysis (89 participants who completed preprogram and 2‐month follow‐up surveys; 76 participants who completed all surveys to examine longer‐term behavioral trends).

Surveyed individuals were highly involved in LFA management on their property and concerned about LFA before the meeting (Table 2). Most individuals in the control (74.7%) and intervention (77.8%) communities reported engaging in ≥3 LFA control actions on their property in the 6 months prior to attending the meeting. Individuals engaged in recruitment and coordination behaviors less often (20.3% of control residents and 19.8% of intervention residents engaged in such behaviors ≥3 times in the same period). Our sample therefore represented model landowners (Ma et al. 2012) who were highly concerned about LFA and had attempted to manage LFA on their property but rarely communicated with their neighbors to manage LFA across property boundaries.

### Intervention Effects on Beliefs of Others

Of the 7 social perceptions examined, the intervention significantly and positively (*p* < 0.05) predicted expected reciprocity. The relationship between the intervention and perceived possibility for reputational sanctions, collective efficacy, and descriptive norms was nearly significant when controlling for preprogram perceptions (Table 3). The intervention did not significantly predict the remaining 3 social perceptions examined. However, the coefficients associated with the intervention were in the direction hypothesized (Table 3). The adjusted estimated effect of the intervention on perceptions of expected reciprocity at the 2‐month follow‐up was 0.72 scale points (Table 3). The intervention also significantly and positively predicted self‐reported knowledge of effective control tactics (Table 3). We obtained similar results for all perceptions with linear regression (Supporting Information). Regression analysis indicated that several of these altered perceptions, including expected reciprocity, predicted recruitment and coordination behavior reported in 2‐ and 7‐month follow‐up surveys (Supporting Information).

**Table 3:** Results of ordinal logistic regressions used to analyze the effect of microinterventions (0, control; 1, intervention) on beliefs^a^ about others during 2‐month follow‐up surveys when adjusting for preprogram beliefs.*b*

	Expected reciprocity	Collective efficacy 1	Collective efficacy 2	Injunctive norms	Descriptive norms	Perceived potential for reputational rewards	Perceived potential for reputational sanctions	Knowledge of control tactics
Intervention	0.722 (0.344)*	0.526 (0.274)^†^	0.442 (0.322)	0.478 (0.395)	0.587 (0.397)†	0.329 (0.310)	0.952 (0.580)^†^	0.851 (0.333)*
Preprogram belief	0.328 (0.255)	0.351 (0.197)^†^	0.501 (0.180)**	0.754 (0.187)**	0.790 (0.277)**	0.307 (0.145)*	0.648 (0.236)**	0.363 (0.104)**
*n*	86	84	84	85	82	85	85	84
*R* ^2^	0.031	0.022	0.045	0.083	0.086	0.019	0.073	0.065
Log likelihood	–136.72	–152.57	–139.83	–126.30	–121.73	–120.95	–84.51	–119.07

a For each belief, we included the preprogram belief as a covariate in case matching and randomization in our experiment did not lead to balancing in the covariate.

^b^Significance: ^**^ *p* ≤ 0.01; ^*^ *p* ≤ 0.05; ^†^ *p* ≤ 0.10.

### Intervention Effects on Recruitment and Coordination Behavior

**Figure 1:**
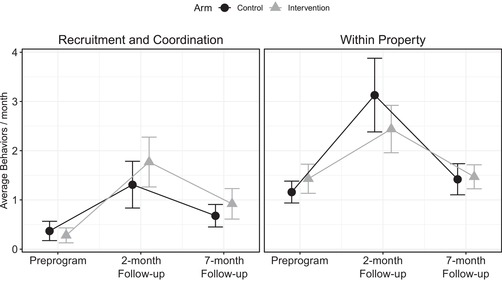
Mean (95% CI) reported number of recruitment and coordination and within‐property behaviors per month in intervention and control communities among those who completed the preprogram survey (n = 89), the 2‐month follow‐up (n = 89), and the 7‐month follow‐up (n = 76).

**Figure 2:**
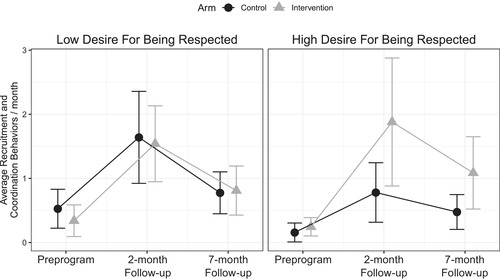
Mean (95% CI) reported number of recruitment and coordination behaviors per month in intervention and control communities in the preprogram surveys and the 2‐ and 7‐month follow‐up surveys by respondent reported desire (high and low) to be respected. Those who more highly desired being respected answered that being respected or held in high regard by others was moderately or very important to them (n = 33), and those who did not highly desire being respected answered that being respected was somewhat or not at all important to them (n = 53).

The intervention did not significantly affect recruitment and coordination behavior reported at the 2‐ and 7‐month follow‐up (Table 4, Fig. 1 & Supporting Information). The estimated rate of recruitment and coordination behavior in the intervention was 1.424 times the rate of the control at 2 months (95% CI 0.90–2.25, *p* = 0.13) and 1.428 times the rate of the control at 7 months (95% CI 0.93–2.21, *p* = 0.11) in the adjusted Poisson analysis. A subsample analysis may explain the lack of a significant difference (Fig. 2).

**Table 4:** Coefficients and cluster robust standard errors (in parentheses) from Poisson regression models analyzing effects of the microinterventions (0, control; 1, intervention) on property level and recruitment and coordination behavior for invasive species management when adjusting for preprogram behavior.^a^

	Full sample				Subsample of those who valued being respected	
	2‐month follow‐up, recruitment and coordination behavior	7‐month follow‐up, recruitment‐and‐ coordination behavior	2‐month follow‐up, within property behavior	7‐month follow‐up, within property behavior	2‐month follow‐up, recruitment and coordination behavior	7‐month follow‐up, recruitment and coordination behavior
Intervention	0.354 (0.233)	0.356 (0.222)	–0.384 (0.175)^b^	–0.049 (0.151)	0.822 (0.123)^a^	0.700 (0.237)^a^
Preprogram engagement in recruitment and coordination behavior	0.074 (0.031)^b^	0.078 (0.024)^a^			0.112 (0.093)	0.161 (0.071)^b^
Preprogram engagement in property‐level behavior			0.059 (0.015)^a^	0.046 (0.012)^a^		
Model fit	adjusted *R* ^2^ = 0.067	adjusted *R* ^2^ = 0.090	adjusted *R* ^2^ = 0.115	adjusted *R* ^2^ = 0.068	adjusted *R* ^2^ = 0.110	adjusted *R* ^2^ = 0.150
*N*	89	76	89	76	33	27

a *p* ≤ 0.01.

b *p* ≤ 0.05.

Including desire for respect by others as an interaction term with the intervention indicated a significant interaction effect on 2‐month recruitment and coordination behavior (*β* = 0.45 [SE 0.22], *p* = 0.04). For the subsample of those who highly desired respect from others (i.e., answered that they thought being respected was “very” or “moderately” important, hereby referred to as “reputationally oriented landowners; *n* = 33), the rate of recruitment and coordination behavior reported in the intervention group was 2.28 times the rate of the control group at 2 months and 2.01 times the rate at 7 months (2‐month incidence rate ratio: 2.28, 95% CI 1.79–2.90, *p* < 0.01; 7‐month incidence rate ratio: 2.01, 95% CI 1.26–3.21, *p* < 0.01) in the adjusted analysis (Table 4). We obtained similar results with negative binomial regression (Supporting Information). Among reputationally oriented landowners, perceived possibility for reputational sanctions at the 2‐month follow‐up significantly predicted 2‐month recruitment and coordination behavior (*β* = –0.428 [SE = 0.143], *p* < 0.01; more negative means more sanctions).

### Intervention Effects on Conservation Action on Property

Intervention participants engaged in fewer within‐property actions at 2 months and a similar amount at 7 months after meetings than the control group (Fig. 1). The intervention significantly and negatively predicted within‐property behavior at 2 months (Table 4). For each within‐property behavior in the control group, there were 0.681 behaviors in the intervention group (incidence rate ratio: 0.681, 95% CI 0.483–0.960, *p* = 0.03). With the negative binomial model, the intervention did not significantly predict 2‐month within‐property behavior (Supporting Information). The intervention was not a significant predictor of within‐property behavior at 7 months with the Poisson (Table 4) and negative binomial models (Supporting Information). The increase in within‐property contributions among those in the control group relative to those in the intervention group was driven primarily by the control group's more frequent use of commercial products to control LFA on their property at the 2‐month follow‐up (Supporting Information). At this follow‐up, those in both the intervention and control groups were, on average, exceeding the recommended use of a commercial product of once every 4–6 weeks (Supporting Information).

## Discussion

Existing private lands conservation programs often reach only a limited subset of highly motivated model landowners already interested in and contributing to conservation. To reach other landowners and enhance effectiveness of highly motivated individuals, conservation outreach could leverage those model landowners’ efforts. Already invested in conservation, model landowners could further share information, recruit, and coordinate with community members, facilitating collective and persistent engagement (Snyder & Broderick 1992; Ma et al. 2012; Graham & Rogers 2017). Motivating such collective actions may require changing those landowners’ beliefs about what others think or do in their community regarding a conservation behavior (Geiger & Swim 2016). We found measurable effects of a series of microinterventions, which can be integrated into existing outreach initiatives. The microinterventions significantly influenced model landowners’ expected reciprocity beliefs, and among a subsample of model landowners concerned with their reputations, they led to approximately twice the rate of recruitment and coordination behavior compared with a knowledge‐transfer approach.

Although our effect sizes were moderate and did not demonstrate significant effects of the intervention on recruitment and coordination for our full sample, our findings have conservation implications. When model landowners are particularly driven by reputational influences, microinterventions may effectively encourage landowners to recruit and coordinate with others in their community. In turn, those collective behaviors may enhance the conservation program’s overall impact (Ma et al. 2012). Our findings suggest social perceptions, especially expected reciprocity, may be important for understanding recruitment and coordination behavior and be altered through interventions that can be easily incorporated into conservation programs. These findings are consistent with Geiger and Swim (2016), who found that altering social perceptions can encourage people to discuss climate change with others.

Our finding regarding the importance and malleability of expected reciprocity is consistent with studies linking perceptions of expected reciprocity to collective environmental behavior (Lubell et al. 2007), environmental policy support (Lubell et al. 2006), and residents’ invasive species control efforts in Hawaii (Niemiec et al. 2016), Australia (Marshall et al. 2016), and Montana (Lubeck 2018). We could not determine whether reciprocity significantly mediated the intervention effects on behavior because of our small sample size. The intervention predicted expected reciprocity at the 2‐month follow‐up, and expected reciprocity at 2 months predicted recruitment and coordination behavior at the 2‐ and 7‐month follow‐ups. Thus, a potentially promising line of inquiry may be to explore whether expected reciprocity mediates the impacts of microinterventions on recruitment and coordination behavior.

Further research is needed on how changing social perceptions motivates recruitment and coordination behavior; our estimates of the power of the microinterventions at changing those behaviors may be conservative. We did not include a true control (i.e., no meeting). Moreover, the knowledge‐transfer (control) meetings were relatively effective at increasing recruitment and coordination behavior. One of the reasons the knowledge‐transfer control meetings may have been so effective was that residents in both meetings were offered an incentive for recruiting and coordinating with neighbors (i.e., BIISC staff would help residents apply a free application of pesticide if they recruited enough neighbors). Another reason our estimates of the power of the microinterventions at changing recruitment and coordination behaviors may be conservative is that before initiation of the outreach programs, self‐reported social perceptions were relatively high and knowledge was relatively low. In situations where knowledge is high but perceived norms, reciprocity, and reputational incentives are low, the microinterventions may have larger effect sizes than knowledge‐transfer approaches.

Our finding that reputational concern moderated the intervention effects is consistent with studies suggesting that some individuals may be more reputationally driven than others (Simpson & Willer 2008). For those individuals, enhancing the visibility of contributions appeals to their reputational concerns and thus increases their contributions to public goods (Simpson & Willer 2008). Our results highlight the importance of examining the interactions between conservation‐behavior‐related interventions and individual values and characteristics (Simpson & Willer 2015). Investigating such interaction effects may help organizations deliver different interventions depending on community context to more effectively target populations based on their characteristics.

Model landowners given the intervention engaged in slightly fewer within‐property conservation behaviors at 2 months than those in the control group. However, at that time, model landowners in both control and intervention communities were, on average, exceeding the recommended frequency of commercial pesticide application. One likely explanation for this is that after meetings, those in the intervention redirected their efforts away from excessive use of pesticide on their property toward recruiting neighbors to coordinate their efforts in monthly applications, which the BIISC outreach coordinator emphasized was a more effective strategy. Those from control communities may have felt motivated to do more to control LFA but did not feel comfortable approaching neighbors. Instead, they increased their use of a commercial pesticide on their property right after the meeting without trying to coordinate with neighbors. This explanation is consistent with the spike in within‐property contributions occurring primarily as a result of a difference in use of a commercial product, which BIISC emphasized residents should apply once a month at the same time as neighbors to be most effective. The BIISC found that in the year following intervention and control outreach programs, more neighborhood groups from intervention communities contacted them asking for help running coordinated neighborhood‐scale pesticide application for LFA.

Another potential explanation for the temporary spike in within‐property contributions among the control relative to the intervention is that the model landowners receiving the intervention felt they had fulfilled their obligation to the collective or used up their time or energy by recruiting others. They may have not felt obligated or had the time or energy to invest as much effort on their property. If true, this may imply a trade‐off between motivating recruitment and coordination and motivating within‐property contributions.

Key limitations of our study were our focus on self‐reported behavior and that we did not obtain data on LFA populations. Many model landowners did not remain enrolled in our experiments over time, which may have led to less precise estimates. Furthermore, the model landowners who remained enrolled were slightly more engaged in property‐level action than those who dropped, suggesting our findings may apply only to more engaged landowners (Supporting Information). Finally, we only tracked behavior at 2 and 7 months following the outreach program. Successful conservation often requires sustained action for a long period (Dayer et al. 2018). Controlling LFA, for example, requires monthly applications of pesticides for a year and continuous monitoring for LFA in perpetuity. Recruitment and coordination and property‐level behaviors were lower at 7 months than 2 months after the outreach meetings. At 7 months, property‐level behaviors returned to preprogram levels.

The intervention and past behavior explained only a small portion of the overall variance in recruitment and coordination behavior at 2‐ and 7‐month follow‐ups. This finding suggests there are additional factors influencing landowners’ willingness to reach out to others that could inform intervention strategies. For example, some key barriers preventing landholders from working across boundaries for invasive species control may be a culture of landowner independence and the belief that other landowners value invasive species because they provide services (e.g., privacy) (Ma et al. 2018). Landholders’ unwillingness to work with their neighbors on management of invasive species may be due to landholder's beliefs that collective action may be ineffective and that others are too busy, are physically incapable, and prefer to work alone (Marshall et al. 2016).

Shifting social perceptions regarding LFA management may be easier than shifting social perceptions regarding controversial conservation problems. For conservation problems in which stakeholders have well‐known, diverse, and opposing perspectives, model landowners who attend a meeting may assume those at the meeting do not represent the broader community. The microinterventions under such circumstances might have a lesser effect or no effect on their social perceptions regarding the community at large.

In our study, model landowners and others possessed relatively high levels of knowledge about LFA, there were effective, discrete, and time‐bound management options, and LFA was causing significant socioeconomic impacts. In other contexts, our microinterventions may need modification, depending on the conservation problem and target population. Options include coupling microinterventions with activities such as participatory mapping (Ravnborg & Westermann 2002) or collective learning and experimentation (Graham & Rogers 2017) to ensure model landowners have sufficient knowledge of the problem and potential solutions to feel comfortable approaching others (Geiger et al. 2017).

Our study adds to knowledge of the social dimensions of private lands conservation (e.g., Moon & Cocklin 2011; Ma et al. 2012; Marshall et al. 2016). In particular, our results suggest model landowners who are concerned about their reputations can be encouraged to recruit and coordinate with others for conservation causes. Model landowners recruiting and coordinating with neighbors could expand the reach, uptake, and effectiveness of private‐lands conservation worldwide.

## Supporting information

A description of the implementation of intervention components (Appendix S1), the recruitment of study participants (Appendix S2), analysis methods (Appendix S3), detailed additional results (Appendix S4), additional figures and tables (Appendix S5), and additional literature cited (Appendix S6) are available online. The authors are solely responsible for the content and functionality of these materials. Queries (other than absence of the material) should be directed to the corresponding author.
