# Supplementary material for: Motivating landowners to recruit neighbors for private land conservation
Source: Conserv Biol. 2019 Feb 20;33(4):930–41. doi: 10.1111/cobi.13294 (PMC6850448; doi:10.1111/cobi.13294)
Supplement: Supplementary file 1 — A description of the implementation of intervention components (Appendix S1), the recruitment of study participants (Appendix S2), analysis methods (Appendix S3), detailed additional results (Appendix S4), additional figures and tables (Appendix S5), and additional literature cited (Appendix S6) are available online. The authors are solely responsible for the content and functionality of these materials. Queries (other than absence of the material) should be directed to the corresponding author. [file COBI-33-930-s001.docx]

**APPENDICES**

**S-1: IMPLEMENTATION OF INTERVENTION COMPONENTS**

At both intervention and control community meetings, BIISC’s outreach director gave residents a slide-show lecture about the threat posed by LFA as well as how to implement proper LFA management techniques. Residents in both intervention and control community meetings were also informed that they were eligible to receive a free LFA Demonstration Day from BIISC; if accepted, on that Demonstration Day, BIISC staff would help residents apply a free application of pesticide. Residents were eligible to receive these Demonstration Days if they recruited several of their neighbors to commit to attending a Demonstration Day and followed up with BIISC to schedule a date and location in their neighborhood to host the Demonstration Day. Residents in both control and intervention communities thus were provided with information on LFA impacts and LFA management techniques as well the opportunity for subsidized control supplies.

In intervention communities, we added the intervention components of communication, goal-setting, increased visibility of efforts, and public commitment-making among residents. First, we asked residents to engage in a discussion with their neighbors about their past experience dealing with LFA. We then asked participants to report findings from their discussion back to the group, and we recorded their responses on a white board. After residents were informed about effective LFA control techniques, we asked residents to engage in a group goal-setting process with their neighbors. During this process, they identified an achievable collective LFA control goal for the following three months; we emphasized that the goal did not have to be eradicating LFA, but it could be focused, rather, on educating several of their neighbors or on surveying all properties on their street.

After neighborhood groups developed a goal, we asked them to share their agreed-upon goal with the others in the room. We then asked them to commit publicly (by raising their hand) to work toward the agreed-upon collective goals and/or engage in some other action(s) to control LFA in the next three months. We encouraged participants to share these commitments with others who could not attend the meeting by signing a community poster about LFA control efforts that would be displayed in a local public space, such as a community association or school. We also asked residents to share their efforts by putting up a yard sign on their property, which read, “LFA is being controlled here” and handing out informative post-cards to other residents. On one side, the cards included instructions on how to survey for LFA; on the other side, the cards included a description of their neighborhood goal and how to become involved in neighborhood LFA control efforts.

The day after the community meeting, researchers followed up via email with each group of neighbors from intervention meetings to remind the participants of their group goal. To enhance communication, researchers also invited each participant in the intervention communities by email to a LFA management Facebook page specific to their community. On that page, a researcher posted a photo of the signed community poster and all documents from the meeting. On the Facebook page, researchers also provided periodic reminders to residents to contact their neighbors and engage in one or more of the actions they pledged to do.

**S-2: RECRUITMENT OF STUDY PARTICIPANTS**

In assigning communities to the control condition versus the intervention condition, we sought, wherever possible, to balance the key characteristics of median income, age, the extent of vacant lots, the level of urbanity (e.g., whether the community was rural, peri-urban, or urban), and the existence of formal organizations within the community. We matched based the age of the population and the income of the population because these variables that have been suggested to influence beliefs about others in one’s community, such as collective efficacy beliefs (Sampson et al. 1997). We matched based on the number of vacant lots, because studies have linked vacant lots with enhanced spread of invasive species (Niemiec et al. 2018), which could reduce landowner’s collective efficacy beliefs regarding their ability to control these species (Yung et al. 2016). We also matched based on the level of urbanity, because studies have found that in rural areas especially, there are often norms against cooperation among neighbors (Ravnborg and Westermann 2002). Finally, we matched based on the existence of formal organizations (i.e. community associations) for two reasons. First, formal organizations can help facilitate community interaction, which studies have linked with enhanced community collective action in response to environmental threats (Flint and Luloff 2007). Second, BIISC’s previous outreach efforts regarding invasive species control had typically been run through existing community associations. Thus, the presence of a community association may have influenced the community’s exposure to previous outreach programs focused on invasive species control.

Once we carried out the experiment, we replaced one control community due to low resident turnout. The replacement community was one that had enrolled after the initial program deadline. Table A1 shows overall levels of characteristics for communities assigned to the two conditions after the replacement.

For both control and intervention communities, we began recruitment for the community meeting between one and three weeks in advance of the meeting date. Recruitment strategies included: distributing meeting information and invitations via community email lists and community-association websites; placing hangers on residents’ doors; and handing out flyers at public locations (such as community mailboxes). Prior to each meeting, we contacted community leaders to discuss appropriate methods for each community; this sensitivity to the micro-localities led to some variation in recruitment among the different communities. For example, while some leaders reported that door-hangers would be an effective strategy for notifying neighbors, others in more rural locations indicated that approaching people’s homes might be dangerous due to, for example, loose dogs. They also suggested that doing so might violate neighborhood social norms related to privacy, for example.

Upon arriving at the community meeting, we informed participants that we were conducting a study on residents’ experiences with LFA. At that time, we invited them to enroll in the study and complete a survey before the meeting started. We then mailed (or emailed) follow-up surveys two months and seven months after the meeting to residents who completed the pre-program survey (See Table A2 and below for a description of the survey). We did not inform residents about the different techniques that we were using at meetings in other communities. We provided respondents with $5 gift cards once they completed the pre-treatment survey; another $5 gift card upon completing the 2-month follow-up survey, and a $10 follow-up gift card upon completing the 7-month follow-up survey. The institutional review board from the primary university approved the informed consent process described herein.

**S-3. ANALYSIS METHODS**

Our analysis took place in several phases. First, we conducted an unadjusted analysis of all behavioral outcomes, in which we compared the mean recruitment and coordination and within-property behavior between residents given the intervention and control at 2- and 7-month follow-ups. For the unadjusted analysis, we used a Mann-Whitney U test, given that our behavioral outcomes were count data and not normally distributed. We also conducted an adjusted analysis of our primary behavioral outcomes using generalized linear regression models. In the regression models, we adjusted for the covariate of pre-meeting engagement in LFA control behavior, as randomization did not fully balance this co-variate (Table 2). In all regression analyses, we accounted for potential non-independence among participants in the same community by using robust standard errors clustered at the community level.

Because our behavioral variables were count outcomes and thus should not necessarily be modeled using linear regression, for the adjusted analyses we selected the family of regressions with the lowest 10-fold cross-validated mean-squared error (MSE). We estimated MSE from linear, Poisson, negative binomial, and zero inflated Poisson models. The Poisson regression had the lowest MSE for modeling both recruitment and coordination behavior and within-property behavior at the first time period (2 months), so we used Poisson regression in subsequent analyses. We also conducted our analysis using negative binomial regression to assess the sensitivity of the study results to the use of qualitatively different models. Negative binomial regression is a commonly used model for overdispersed count data.

We also conducted an analysis of perceptions of others (i.e. norms, reciprocity, collective efficacy, reputational incentives). We first conducted unadjusted Mann-Whitney U tests to examine which perceptions differed significantly between control and intervention communities at two months after experimental meetings. We then used ordinal logistic regression to examine whether the intervention predicted post-meeting perceptions after controlling for pre-meeting perceptions in case randomization did not lead to balancing in these co-variates. We also conducted a sensitivity analysis using linear regression. Finally, we conducted a moderation analysis to examine whether the intervention effects differed based on respondents’ personal norms and value orientations. To do so, we examined whether the interaction between personal norms or value orientations and the intervention was a significant predictor of post-meeting behavior.

Attrition rates, while high, were similar for control and intervention communities (Table 2). Since our attrition rates were close to 50% between the pre-program and 2-month follow-up surveys, and multiple imputation can lead to biased results with such high levels of missingness (Leite and Beretvas 2010), we did not use multiple imputation for our analyses. Rather, we conducted our 2-month follow-up survey analysis only with residents who completed 2-month post-surveys (n=89). We focused our analysis of the 7-month follow-up data on those who had completed both the 2- and 7- month follow-up surveys (rather than adding in those who completed the 7- but not 2- month follow-up) because collecting data at the 7-month mark was primarily meant to examine whether the 2-month behavioral trends persisted over time. We did not want observed trends over time to be the result of new individuals being added to the sample.

To examine whether the high levels of attrition led to differential loss to follow-up, we examined whether mean pre-meeting behaviors, perceptions, and demographics changed between control and intervention groups as the same sample size decreased for each subsequent survey due to attrition (Table A3). While there was a slight initial unbalance in some characteristics at the pre-survey, attrition did not appear to differentially affect mean pre-meeting characteristics among control and intervention groups (Table A3).

We also conducted Mann-Whitney U tests to examine whether there were significant differences in pre-meeting recruitment and coordination behavior between those who dropped out after the pre-survey and those who stayed in the study to complete the 2-month follow-up and the 2 and 7-month follow-up in the intervention and control arms. We conducted statistical tests only for pre-meeting recruitment and coordination behavior because this was our primary outcome of interest. Furthermore, conducting statistical tests for all baseline characteristics outlined in Table A3 would likely lead to significant results for some variables due to chance.

There were no significant differences in pre-meeting recruitment and coordination between those who dropped out after the pre-survey and those stayed in the study to complete the 2-month follow-up in the intervention (Mann Whitney U Test: Mean drop out=1.44, Mean remaining=1.70 ; z=-0.672, p=0.50) and control groups (Whitney U Test: Mean drop out =1.38, Mean remaining=2.24; z=-0.920, p=0.36). There were also no significant differences in pre-meeting recruitment and coordination between those who dropped out at some point and those stayed in the study to complete the 2- and 7-month follow-up in the intervention (Whitney U Test: Mean drop out=1.31, Mean remaining=1.92, z=-0.861, p=0.39) and control groups (Whitney U Test: Mean drop out=1.32, Mean remaining=2.39; z=-0.979, p=0.33).

In addition to examining differential loss to follow-up, we also examined whether our overall sample enrolled in the entire experiment changed as a result of attrition. Table A3 suggests a trend towards more engaged individuals staying in the study. Those who stayed in the experiment for the 2 month study after the pre-survey were marginally significantly more engaged in LFA management on their property than those who did not (Mann-Whitney U test: Mean drop out = 6.479, Mean remaining = 7.831; z =-1.949, p =0.051). However, there were no significant differences in pre-meeting recruitment and coordination among those who stayed in the study to complete the 2-month follow-up and those who dropped out after the pre-survey (Mann-Whitney U test: Mean drop out = 1.41, Mean remaining = 1.96; z =-1.123, p =0.26). There were also no significant differences in pre-meeting recruitment and coordination between those who dropped out at some point and those stayed in the study to complete both the 2- and 7-month follow-ups (Mann Whitney U test: Mean drop out = 1.31, Mean remaining = 2.16; z=-1.309, p=0.19).

Our analysis of the effects of attrition therefore suggests that attrition did not lead to differential loss to follow-up overall; thus, our estimates of the effect of the intervention compared to the control were not biased by the high rates of attrition. However, we did find some evidence that the remaining individuals in our study sample may have been more engaged in property-level action, which could influence the generalizability of our conclusions. In particular, these results suggest that our study findings may only apply to those individuals who are more engaged on their property.

**S-4: DETAILED ADDITIONAL RESULTS**

*Unadjusted Effects of Intervention on Social Perceptions*

In an un-adjusted analysis of all hypothesized perceptions at 2-months, the mean expected reciprocity and the perceived possibility for reputational sanctions were significantly different between the control and the intervention groups (Expected Reciprocity: Mean Difference (Intervention-Control)= 0.670, z=-2.391, p=0.02, n=88; Reputational Sanctions: Mean Difference (Intervention-Control)= 0.422, z= 2.012, p=0.04, n=87). Mean perceived knowledge of effective control tactics and collective efficacy perception 1 (“together residents can make a difference”) were marginally significantly different between control and intervention (Knowledge: Mean Difference (Intervention-Control)= 0.656, z=-1.798, p=0.07, n=88; Collective Efficacy: Mean Difference (Intervention-Control)= 0.578, z=-1.579, p=0.114, n=88).

*Unadjusted Effects of Intervention on Recruitment and Coordination Behavior*

The difference in mean recruitment and coordination behavior between intervention and control groups was not significant at 2 and 7 months in the unadjusted analysis (Mann-Whitney U test; 2 months: Difference in mean behavior/month (intervention-control) =0.460, z=-1.67, p=0.10, n= 89; 7 months: Difference in mean behavior/month (intervention-control) =0.242, z=-0.927, p=0.354, n=76).

*Effects of Social Perceptions on Recruitment and Coordination Behavior*

We conducted Poisson regression analysis to examine whether any of the perceptions changed by the intervention predicted 2- and 7- month recruitment and coordination behavior. We could not, however, conduct a full mediation analysis to examine whether the various social perceptions changed by the intervention were responsible for the changes in behavior, given that the intervention did not significantly predict post-meeting recruitment and coordination behavior among the full sample. Poisson regressions revealed that among the full sample, expected reciprocity, collective efficacy 1, and descriptive norms reported in 2-month follow-up surveys significantly predicted 2-month recruitment and coordination behavior when controlling for pre-program behavior (Expected Reciprocity: β= 0.234, Cluster Robust SE=0.081, p<0.01; Collective Efficacy 1: β= 0185, Cluster Robust SE= .061, p<.01; Descriptive Norms: β = 0.233, Cluster Robust SE= 0.095, p=0.015). Expected reciprocity also significantly predicted 7-month recruitment and coordination behavior when controlling for pre-program behavior (β=0.294, Cluster Robust SE= 0.119, p=0.014). Poisson regressions also revealed that, among the smaller sample of residents who highly valued being respected, 2-month perceived possibility for reputational sanctions significantly predicted 2-month post-meeting recruitment and coordination behavior (β= -0.428, Cluster Robust SE= 0.143, p<0.01; note: more negative means more sanctions).

*Unadjusted Effects of Intervention on Conservation Behavior on Property*

Surprisingly, the mean property-level behavior was greater for the control group at 2 months after the community meetings; however, this difference was not significant in the unadjusted analysis using a Mann Whitney U test (Difference in mean behavior/month (intervention-control) =-0.69, .z=0.905, p=0.37, n=89; Figure 1). At 7 months following the community meetings, mean property-level behavior converged and the mean differences between intervention and control remained insignificant in the unadjusted analysis (difference in mean behavior/month (intervention-control) =0.050, z=-0.746, p=0.46, n=76).

*Moderators of Intervention Effects on Recruitment and Coordination Behavior*

Our selection of potential moderators drew on recent social psychology literature suggesting that social influence approaches such as our micro-interventions are more effective at motivating a subset of the population with certain values and beliefs (Schultz et al 2016; Simpson and Willer 2008). Recent studies have provided preliminary evidence that social perceptions may have a greater impact on more egoistic individuals, who lack the strong intrinsic motivation that altruists may have for contributing to a public good and who may care more about their reputation (Simpson and Willer 2008). Studies also suggest that social mechanisms may have a greater impact on individuals who do not already have strong personal norms around an issue (Schultz et al. 2016). Schultz et al. (2016) define personal norms as a “person’s internal standards for conduct associated with a particular behavior that derive from internalized values.” The Elaboration Likelihood Model suggests that normative information may have a greater impact on individuals with low personal norms because such individuals will be more likely to make decisions based on external cues, such as norms, rather than careful and deliberate consideration based on values and attitudes (Schultz et al. 2016). We therefore measured residents’ personal norms and value orientations to examine whether these factors moderated the impact of the social influence intervention.

We tested four measures of egoism as moderators of the intervention effects (Table A2: Valuing Being Respected, Valuing Wealth, Valuing Being Influential, Valuing Authority). The interaction term between “Valuing Being Respected” and the intervention was significant, indicating that Valuing Being Respected was a significant moderator of the intervention effects at 2 months (Poisson Regression: β= .450, Robust SE= .216, p=.038; Negative Binomial Regression: β= .548, Robust SE=.237, p=.021). Though the associations between valuing being respected and the other egoistic social value orientations were positive and significant (r_s_= .22 for valuing wealth, r_s_=.38 for valuing being influential, r_s_= .38 for valuing authority), we did not find evidence that any of these other egoistic social value orientations were significant moderators of the intervention effects on behavior (Valuing Wealth Interaction Term: Poisson Regression: β=-0.12 , Robust SE=0.317, p=0.704; Valuing Being Influential Interaction Term: Poisson Regression: β=0.147, Robust SE= 0.250, p=0.557; Valuing Authority Interaction Term: Poisson Regression: β=-0.225, Robust SE= 0.180 , p=0.212). We also found no evidence that personal norms was a significant moderator of the intervention effects (Personal Norms Interaction Term: Poisson Regression: β=0.213, Robust SE=0.333, p=0.523).

**S-5: ADDITIONAL FIGURES AND TABLES**

**Table A1**: Characteristics used to match 10 communities to control and intervention groups and the extent of overall balancing in community characteristics between the 5 communities assigned to intervention and the 5 communities assigned to control (after one initial control community was dropped and replaced). Data on median income, absentee lots, and percent of residents age 65 or older was obtained from the 2015 American Community Survey from Simply Map.

| **Community Characteristic used in Matching** | **Control** | **Intervention** |
| --- | --- | --- |
| Median household income | Average of $47,852 among 5 communities | Average of $55,678 among 5 communities |
| Rural or urban | 1 rural, 3 peri-urban, 1 urban (in Hilo) | 1 rural, 3 peri-urban, 1 urban (in Hilo) |
| Percent of residents 65 or older | Average of 17% | Average of 15% |
| Percent vacant lots | Average of 18% | Average of 15% |
| Presence of community association | Three of the five communities had active community association | Two of the five communities had active community association |

**Table A2**: Behaviors and perceptions measured in pre-program and 2-month follow-up surveys. The following perceptions were measured because previous literature suggested they could mediate, moderate, or confound the effects of the intervention on self-reported behavior.

| **Construct** | **Why Variable was Included** | **Survey Item** | **Adapted from** |
| --- | --- | --- | --- |
| Behavioral Outcome: Recruitment-and -Coordination Behavior | Primary Behavioral Outcome | In the past six months, how often have you….   1. Given information to someone else in your community about how to control or survey for LFA? 2. Spoken to or written a letter to someone else in your community to convince them to control LFA? 3. Organized efforts with residents in your community to control LFA?   (Circle one: Once, twice, three times, four times, five times, six or more times) | Behavioral Outcome: Recruitment-and -Coordination Behavior |
| Behavioral Outcome: Within-Property Conservation Action^[[1]](#footnote-1)^ | Secondary Outcome | In the past six months, how often have you….   1. Surveyed your property to see if you have LFA? 2. Used any commercial product intended for insect control to manage LFA on your property? 3. Used any other substance or product to manage LFA on your property 4. Hired someone to control LFA?   (Circle one: Once, twice, three times, four times, five times, six or more times) |  |
| Descriptive and Injunctive norms | Intervention designed to change | (DESCRIPTIVE) What percentage of residents in your community do you believe have taken actions to reduce the spread of LFA in the past 3 months? (0-10%, 10-25%, 25-50%, 50-75%, 75-90%, 90%-100%)  (INJUNCTIVE) People in my community care about reducing the spread of LFA (Likert Scale) |  |
| Reputational Incentives | Intervention designed to change | (REPUTATIONAL REWARD) How do you think others in your community would perceive you if you were to take action to control LFA on your property and in your community in the next month? (-3 to 3, with -3 being very negative, 0 being no reaction, and 3 being very positive reaction)  (REPUTATIONAL SANCTIONS) How do you think others in your community would perceive you if you DID NOT take action to control LFA on your property and in your community in the next month? (-3 to 3, with -3 being very negative, 0 being no reaction, and 3 being very positive reaction) | Simon et al. (1998) |
| Expected Reciprocity | Intervention designed to change | My actions to reduce the spread of LFA will motivate others in my community to do the same. (Likert Scale) | Lubell et al. 2007; Kellstedt et al. 2008 |
| Collective Efficacy | Intervention designed to change | A sufficient number of residents can be mobilized to reduce populations of LFA in my community (Likert Scale)  Together, residents could achieve a collective LFA control goal in my community in the next three months (Likert Scale) | Simon et al. 1998 |
| Risk (individual and collective) | Co-variate | How serious of a threat do you believe LFA poses to Hawaii’s ecosystems? (5 pt threat scale)  How serious of a threat do you believe LFA poses to the Hawaiian economy and way of life? (5 pt threat scale)  How serious of threat do you believe LFA poses to your or your pets’ safety or well-being? (5 pt risk scale)  How serious of threat do you believe LFA poses to agriculture in Hawaii? (5 pt risk scale) | Roser-renouf and Nisbet 2008 |
| Personal Norm | Co-variate or Potential Moderator | Taking action to control LFA is the right thing to do (7 pt Likert scale)  I feel a personal obligation to take action to reduce LFA (7 pt Likert scale) | Stern et al. 1999 |
| Knowledge on control tactics | Co-variate | I have the knowledge to control LFA safely and effectively (7 pt Likert scale) | Niemiec et al (2016) |
| Values (Biospheric, Egoistic, and Altruistic) | Potential Moderator | Please rate the importance of these 12 values as a guiding principle in your life. Please vary the scores and rate only a few values as extremely important. (-1 opposed to my values, 0 not important, 3 very important)  Egoistic:  1. Wealth: material possessions, money  2. Authority: the right to lead or command  3. Being Influential: having an impact on people and events  4. Being Respected: Being held in high regard by others  Altruistic:  5. Equality: equal opportunity for all  6. Social justice: correcting injustice, care for the weak  7. Helpful: working for the welfare of others  Biospheric:  8. Unity with nature: fitting into nature  9. Protecting the Earth: preserving nature | Adapted in part from deGroot and Steg (2008) |

**Table A3:** Attrition rates and how attrition influenced mean sample pre-program characteristics. The mean pre-program characteristics reported are the means for the larger sample who completed the pre-program survey, the smaller sample who completed both the pre-program and the 2-month follow-up survey, and the even smaller sample who completed both the pre-program, 2-month follow-up, and 7-month follow-up survey. Similar mean pre-meeting characteristics among the different samples in the control and intervention suggest that attrition did not lead to differential loss to follow-up.

| **Number of Participants Who Completed Survey** | **Total** | **Control** | **Intervention** |
| --- | --- | --- | --- |
| Pre-survey | 162 | 79 | 83 |
| 2- month follow-up | 89 | 42 | 47 |
| 2- and 7-month follow-up | 76 | 38 | 38 |
| 7-month follow-up | 86 | 44 | 42 |
| **Mean Pre-Program Recruitment and coordination Behavior** |  |  |  |
| Pre-survey | 1.71 | 1.84 | 1.59 |
| 2-month follow-up | 1.96 | 2.24 | 1.70 |
| 2- and 7-month follow-up | 2.16 | 2.39 | 1.92 |
| **Mean Pre-Program Property-Level Behavior** |  |  |  |
| Pre-survey | 7.22 | 6.77 | 7.65 |
| 2-month follow-up | 7.83 | 6.98 | 8.60 |
| 2- and 7-month follow-up | 7.55 | 6.76 | 8.34 |
| **Mean Pre-Program Beliefs about others** |  |  |  |
| *Expected Reciprocity* |  |  |  |
| Pre-survey | 5.18 | 5.00 | 5.36 |
| 2-month follow-up | 5.17 | 4.98 | 5.35 |
| 2- and 7-month follow-up | 5.21 | 4.97 | 5.45 |
| *Collective Efficacy* 1: |  |  |  |
| Pre-survey | 4.97 | 4.90 | 5.03 |
| 2-month follow-up | 5.02 | 4.96 | 5.07 |
| 2- and 7-month follow-up | 5.10 | 5.01 | 5.19 |
| *Collective Efficacy* 2 |  |  |  |
| Pre-survey | 5.24 | 5.28 | 5.20 |
| 2-month follow-up | 5.23 | 5.22 | 5.24 |
| 2- and 7-month follow-up | 5.31 | 5.32 | 5.29 |
| *Injunctive Norms*: |  |  |  |
| Pre-survey | 5.19 | 5.09 | 5.28 |
| 2-month follow-up | 5.19 | 5.08 | 5.28 |
| 2- and 7-month follow-up | 5.14 | 5.03 | 5.24 |
| *Descriptive Norms*: |  |  |  |
| Pre-survey | 2.81 | 2.91 | 2.71 |
| 2-month follow-up | 2.82 | 2.88 | 2.76 |
| 2- and 7-month follow-up | 2.83 | 2.91 | 2.76 |
| *Potential for Reputational Rewards* |  |  |  |
| Pre-survey | 5.55 | 5.49 | 5.62 |
| 2-month follow-up | 5.64 | 5.63 | 5.64 |
| 2- and 7-month follow-up | 5.68 | 5.57 | 5.78 |
| *Potential for Reputational Sanctions* |  |  |  |
| Pre-survey | 3.44 | 3.39 | 3.48 |
| 2-month follow-up | 3.44 | 3.41 | 3.46 |
| 2- and 7-month follow-up | 3.36 | 3.38 | 3.34 |
| **Mean Demographics** |  |  |  |
| *Property Ownership* |  |  |  |
| Pre-survey | 1.14 | 1.14 | 1.15 |
| 2-month follow-up | 1.11 | 1.13 | 1.09 |
| 2- and 7-month follow-up | 1.12 | 1.14 | 1.11 |
| *Income* |  |  |  |
| Pre-survey | 3.26 | 2.93 | 2.98 |
| 2-month follow-up | 2.97 | 2.71 | 3.23 |
| 2- and 7-month follow-up | 3 | 2.67 | 3.38 |
| *Property Size* |  |  |  |
| Pre-survey | 2.73 | 3.05 | 2.44 |
| 2-month follow-up | 3.19 | 3.73 | 2.72 |
| 2- and 7-month follow-up | 3.22 | 3.77 | 2.69 |
| *Education* |  |  |  |
| Pre-survey | 3.45 | 3.42 | 3.49 |
| 2-month follow-up | 3.56 | 3.50 | 3.62 |
| 2- and 7-month follow-up | 3.56 | 3.46 | 3.67 |
| *Age* |  |  |  |
| Pre-survey | 59.71 | 57.02 | 62.08 |
| 2-month follow-up | 61.49 | 57.46 | 65.12 |
| 2- and 7-month follow-up | 61.18 | 57.79 | 64.67 |

**Table A4**: Unadjusted mean changes in perceptions in control and intervention communities between pre-program and 2-month follow-up surveys (n=89).

| **Perception** | **Number of Scale Points in Measurement** | **Control**  **Mean Difference (Post-Pre)** | **Control Standard Deviation Difference (Post-Pre)** | **Intervention Mean Difference (Post-Pre)** | **Intervention Standard Deviation Difference (Post-Pre)** |
| --- | --- | --- | --- | --- | --- |
| Expected Reciprocity | 7 pt Likert | -.293 | 1.601 | .021 | 2.059 |
| Collective Efficacy 1: Enough residents can be motived to reduce LFA populations | 7 pt Likert | -.579 | 1.553 | -.185 | 1.913 |
| Collective Efficacy 2: Together residents can achieve a collective goal | 7 pt Likert | -.436 | 1.803 | -.089 | 1.742 |
| Injunctive Norms: Others care about LFA control | 7 pt Likert | -.051 | 1.486 | .239 | 1.32 |
| Descriptive Norms: Prevalence of LFA management behavior in community | 6 pt scale, ranging from 0-100% | -.225 | 1.149 | .345 | 1.407 |
| Potential for Reputational Sanctions (more negative means more potential for sanctions) | 7 pt scale ranging from  -3 (negative reaction to 3 (positive reaction) | .293 | 1.346 | -.136 | 1.047 |
| Potential for Reputational Rewards (more positive means more potential for rewards) | 7 pt scale ranging from  -3 (negative reaction to 3 (positive reaction) | -.146 | 1.590 | .250 | 1.626 |
| Knowledge | 7 pt Likert | 1.650 | 2.058 | 2.09 | 1.938 |
| Threat Perceptions | 5 pt threat scale | .359 | .665 | .391 | .686 |

**Table A5:** Coefficients and cluster-robust standard errors from a series of linear regressions predicting to various perceptions associated with LFA when adjusting for pre-program perceptions (** p=<01, *p=<.05, p=<.10).

|  | Expected Reciprocity | Collective Efficacy 1: Sufficient numbers of residents can be mobilized | Collective Efficacy 2: Together residents can achieve a collective goal | Injunctive Norm: People care about LFA control | Descriptive Norms: Prevalence of LFA behavior in community | Perceived Potential for Reputational Rewards | Perceived Potential for Reputational Sanctions | Knowledge of Control Tactics |
| --- | --- | --- | --- | --- | --- | --- | --- | --- |
| Intervention (0= control, 1= intervention) | .554(.234)* | .490(.233)+ | .387(.282) | .392(.311) | .388(.236)+ | .307(.227) | -.411(.201)+ | .644(.189)** |
| Pre-Program Perception | .190(.133) | .268(.156) | .389(.168)* | .436(.092)** | .424(.087) | .200(.087)* | .244(.117)+ | .249(.063)** |
| N | 86 | 84 | 84 | 85 | 82 | 85 | 85 | 84 |
| R2 | .086 | .075 | .140 | .200 | .247 | .057 | .110 | .20 |
| F | F(2,9)=11.99 | F(2,9) = 7.75 | F(2,9)= 5.43 | F(2,9)= 19.63 | F(2,9)=45.20 | F(2,9)= 4.12 | F(2,9)=3.53 | F(2,9)=9.45 |

**Table A6:** Coefficients and standard errors from negative binomial regression models analyzing effects of the micro-interventions on recruitment and coordination and within-property behavior for invasive species management when controlling for pre-program behavior. Standard errors were clustered at the community level (** p=<01, *p=<.05, + p=<.10).

|  | **2-month Follow-up Recruitment and coordination Behavior** | **7-month Follow-up Recruitment-and- Coordination Behavior** | **2-month Follow-up Within Property Behavior** | **7-month Follow-up Within Property Behavior** | **2-month Follow-up Recruitment and coordination Behavior AMONG THOSE WHO DESIRED BEING RESPECTED** | **7month Follow-up Recruitment and coordination Behavior AMONG THOSE WHO DESIRE BEING RESPECTED** |
| --- | --- | --- | --- | --- | --- | --- |
| Intervention (0= control, 1= intervention) | .333(.211) | .384(.238) | -.347(.185)+ | -.021(.155) | .817(.127)*** | .667(.280)* |
| Pre-Program Engagement in Recruitment and Coordination Behavior | .077(.038)* | .085(.030)** |  |  | .116(.082) | .158(.072)* |
| Pre-Program Engagement in Within Property Behavior |  |  | .060(.016)*** | .049(.012)*** |  |  |
| Model fit | Adjusted R2 =.024 | Adjusted R2=.027 | Adjusted R2= .051 | Adjusted R2= .030 | Adjusted R2= .04 | Adjusted R2= .04 |
| N | 89 | 76 | 89 | 76 | 33 | 27 |

I

**Table A-7**: Unadjusted mean number of self-reported within property contributions per month to LFA control in the pre-program survey, the 2-month follow-up, and the 7-month follow-up among those in intervention and control groups, sorted by the type of contribution.

|  | **Control** | **Intervention** |
| --- | --- | --- |
| **Surveying Property** |  |  |
| Pre-Program | .548 | .634 |
| 2-month Follow-up | 1.44 | 1.45 |
| 7-month Follow-up | .658 | .753 |
| **Using Commercial Product on Property** |  |  |
| Pre-Program | .647 | .688 |
| 2-month Follow-up | 1.81 | 1.36 |
| 7-month Follow-up | .742 | .763 |
| **Using Non-Commercial Product on Property** |  |  |
| Pre-Program | .325 | .468 |
| 2-month Follow-up | 1.17 | .947 |
| 7-month Follow-up | .458 | .432 |
| **Hiring Someone to Control on Property** |  |  |
| Pre- Program | .270 | .315 |
| 2-month Follow-up | .714 | .680 |
| 7-month Follow-up | .358 | .321 |

**S-6: Additional Literature Cited**

De Groot, J. I., & Steg, L. (2008). Value orientations to explain beliefs related to environmental significant behavior: How to measure egoistic, altruistic, and biospheric value orientations. *Environment and Behavior*, *40*(3), 330-354.

Flint, C. G., & Luloff, a. E. (2007). Community Activeness in Response to Forest Disturbance in Alaska. *Society & Natural Resources*, *20*(5), 431–450. https://doi.org/10.1080/08941920701211850

Kellstedt, P. M., Zahran, S., & Vedlitz, A. (2008). Personal efficacy, the information environment, and attitudes toward global warming and climate change in the United States. *Risk analysis*, *28*(1), 113-126.

Leite, W., & Beretvas, S. N. (2010). The performance of multiple imputation for Likert-type items with missing data. *Journal of Modern Applied Statistical Methods*, *9*(1), 8.

Lubell, M., Zahran, S., & Vedlitz, A. (2007). Collective action and citizen responses to global warming. *Political Behavior*, *29*(3), 391-413.

Niemiec, R. M., Ardoin, N. M., Wharton, C. B., & Asner, G. P. (2016). Motivating residents to combat invasive species on private lands: social norms and community reciprocity. *Ecology and Society*, *21*(2).

Niemiec RM, Asner GP, Brodrick PG, Gaertner JA, & Ardoin NM. (2018). Scale-dependence of environmental and socioeconomic drivers of albizia invasion in Hawaii. *Landscape and Urban Planning* 169: 70-80.

Ravnborg, H. M., & Westermann, O. (2002). Understanding interdependencies: Stakeholder identification and negotiation for collective natural resource management. *Agricultural Systems*, *73*(1), 41–56. https://doi.org/10.1016/S0308-521X(01)00099-3

Roser-Renouf, C., & Nisbet, M. C. (2008). The measurement of key behavioral science constructs in climate change research. *International Journal of Sustainability Communication*, *3*, 37-95.

Sampson, R. J., Raudenbush, S. W., & Earls, F. (1997). Neighborhoods and Violent Crime : A Multilevel Study of Collective Efficacy. *Science (New York, N.Y.)*, *277*(August), 918–923.

Schultz, P. W., Messina, A., Tronu, G., Limas, E. F., Gupta, R., & Estrada, M. (2014). Personalized normative feedback and the moderating role of personal norms: A field experiment to reduce residential water consumption. *Environment and Behavior*, *48*(5), 686-710.

Simon, B., Loewy, M., Stürmer, S., Weber, U., Freytag, P., Habig, C., … Spahlinger, P. (1998). Collective identification and social movement participation. *Journal of Personality and Social Psychology*, *74*(3), 646–658. <https://doi.org/10.1037/0022-3514.74.3.646>

Simpson, B., & Willer, R. (2008). Altruism and Indirect Reciprocity: The Interaction of Person and Situation in Prosocial Behavior. *Social Psychology Quarterly*, *71*(1), 37–52. https://doi.org/10.1177/019027250807100106

Stern, P. C., Dietz, T., Abel, T., Guagnano, G. A., & Kalof, L. (1999). A value-belief-norm theory of support for social movements: The case of environmentalism. *Human ecology review*, 81-97.

Yung, L., Chandler, J., Haverhals, M. (2016). Effective Weed Management, Collective Action, and Landownership Change in Western Montana. *Invasive Plant Science and Management*, *8*(2), 193–202. https://doi.org/10.1614/IPSM-D-14-00059.1

1. For both behavioral outcomes, numbers greater than 6 were counted as 6. Thus, the maximum number of property-level behaviors a resident could engage in was 24 and the maximum number of recruitment and coordination behaviors a resident could engage in was 18. [↑](#footnote-ref-1)
